# Time-Shift Correlation Algorithm for P300 Event Related Potential Brain-Computer Interface Implementation

**DOI:** 10.1155/2016/3039454

**Published:** 2016-08-08

**Authors:** Ju-Chi Liu, Hung-Chyun Chou, Chien-Hsiu Chen, Yi-Tseng Lin, Chung-Hsien Kuo

**Affiliations:** ^1^Department of Internal Medicine, School of Medicine, College of Medicine, Taipei Medical University, Taipei 110, Taiwan; ^2^Division of Cardiology, Department of Internal Medicine, Shuang Ho Hospital, New Taipei City 235, Taiwan; ^3^Department of Electrical Engineering, National Taiwan University of Science and Technology, Taipei 106, Taiwan; ^4^Graduate Institute of Biomedical Engineering, National Taiwan University of Science and Technology, Taipei 106, Taiwan; ^5^Department of Biomedical Engineering, National Defense Medical Center, Taipei 114, Taiwan

## Abstract

A high efficient time-shift correlation algorithm was proposed to deal with the peak time uncertainty of P300 evoked potential for a P300-based brain-computer interface (BCI). The time-shift correlation series data were collected as the input nodes of an artificial neural network (ANN), and the classification of four LED visual stimuli was selected as the output node. Two operating modes, including fast-recognition mode (FM) and accuracy-recognition mode (AM), were realized. The proposed BCI system was implemented on an embedded system for commanding an adult-size humanoid robot to evaluate the performance from investigating the ground truth trajectories of the humanoid robot. When the humanoid robot walked in a spacious area, the FM was used to control the robot with a higher information transfer rate (ITR). When the robot walked in a crowded area, the AM was used for high accuracy of recognition to reduce the risk of collision. The experimental results showed that, in 100 trials, the accuracy rate of FM was 87.8% and the average ITR was 52.73 bits/min. In addition, the accuracy rate was improved to 92% for the AM, and the average ITR decreased to 31.27 bits/min. due to strict recognition constraints.

## 1. Introduction

Brain-computer interfaces (BCIs) are systems that interpret the electrical activities of a subject's brain when in command of external devices. Thus, BCIs provide subjects with a nonmuscular method to connect with world. For disabled people who have suffered from spinal cord injuries and strokes, BCIs can improve their independence in their daily lives. In addition, several works have proved that there is a great potential to develop BCI in wider applications, such as robotics [1] and rehabilitation [2]. EEG and hemodynamic monitoring are two different methods to monitor the brain's activities. However, EEG is currently the most common method to obtain brain activity. It has the advantages of low cost, acceptable temporal resolution, high mobility, and higher acceptance by subjects. Moreover, EEG includes noninvasive and invasive recording methods. Noninvasive recording methods are easier to set up. However, noisy signals and low signal amplitude and spatial resolution present challenges in signal processing. Compared to noninvasive approaches, invasive methods require the insertion of microelectrode arrays into subjects' skulls. Invasive recording methods feature the advantages of high accuracy and resolution but are accompanied by health risk considerations.

In robotic applications, integration of BCIs has attracted great attention. A BCI allows subjects to manipulate mobile robots, robotic exoskeletons, and robotic wheelchairs via their “minds.” For example, a brain-actuated wheelchair is a solution for subjects who are unable to use conventional interfaces due to motor disabilities but able to issue commands using their thoughts. Long et al. proposed a hybrid BCI integrating motor imagery-based mu rhythm and P300 potential to control wheelchairs [3]. Subjects were able to command directions by using left and right hand imageries and change speeds via flashing buttons on a graphical user interface. Despite commanding the steering of wheelchair directly, Iturrate et al. used an autonomous navigation system to drive a wheelchair in an indoor environment [4]. Subjects were able to move to defined locations through attending to target stimuli on a panel. Gandhi et al. used an intelligent adaptive user interface to address multiple motion command options for mobile robots [5]. This novel interface offered multiple control options for robotic devices by providing a continuously updated prioritized list as well as improving ITR.

With respect to humanoid robot applications, Chae et al. proposed a BCI to control a humanoid robot navigating in an indoor maze [6]. The power spectral analysis of EEG was used to recognize subjects' intentions. Experiment results reported that all subjects finished navigation tasks successfully. Güneysu and Akin developed a BCI which allowed subjects to interact with a humanoid robot [7]. A portable EEG recorder was used for EEG measurements. Steady-state visually evoked potential (SSVEP) was elicited through a number of flickering stimuli. According to the dominant response frequency of EEG, the humanoid robot was capable of performing defined tasks. Humanoid robots have structures that are similar to those of humans, so they exhibit highly adaptive mobility in uncertain environments, such as walking on rough terrain or encountering sudden disturbances. Humanoid robots therefore show great potential for performing complicated tasks in living and industrial environments. Therefore, to control a humanoid robot with BCI is a promising solution in the future daily life.

P300, which is elicited after a visual stimulus, is detected as a positive deflection in voltage with a latency of around 300 milliseconds. It was reported by Sutton et al. in 1965 [8]. Generally, it occurs most strongly in the parietal and occipital lobes [9]. A P300-based BCI requires a small amount of a subject's data for training and modeling and is feasible for practical applications without requiring long-term training [10, 11]. Rakotomamonjy and Guigue proposed an ensemble of classifiers approach to improve the performance of SVM classifier for BCI application [12]. A small part of dataset was used to train liner SVM through channel selection procedure. The proposed approach addressed the problem of subjects' variabilities responding to visual stimuli. Dal Seno et al. used error potentials (ErrPs) to enhance the performance of P300-based BCI [13]. The genetic algorithm was used as the BCI classifier. By adding ErrP detections, the genetic algorithm (GA) based classifier provided high performance in detecting P300. A P300 speller based on an oddball paradigm allows a subject to communicate a series of letters to a computer [14, 15]. Kaper et al. proposed a P300 speller paradigm based on a support vector machine (SVM) classifier [16]. Their SVM-based BCI system achieved 0.0% error rate when the collected EEG signals were applied with averaging five epochs. The oddball paradigm that is presented to a subject intensifies randomly in rows and columns of a matrix. Each cell contained in the matrix represents a particular letter. Subjects are required to focus on a cell, and the selected row and column elicit P300. BCI classifiers are used to recognize either target or nontarget stimuli according to extracted epochs [17]. Then, the target letter is detected. Usually, a P300-based BCI needs a number of stimuli that flash repeatedly to achieve high accuracy performance. The number of repetitions leads to a trade-off between ITR and accuracy, which has been widely discussed for P300-based BCIs [18].

**Figure 1:**
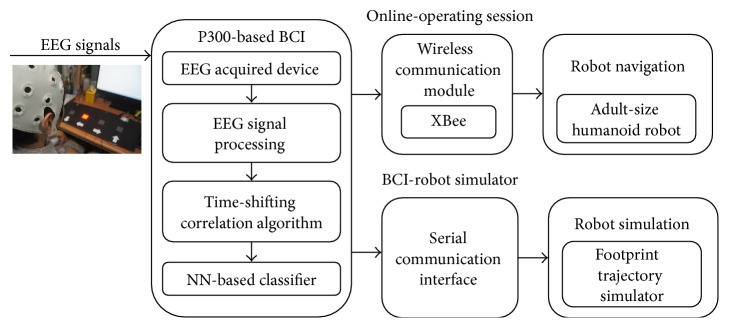
The proposed system architecture.

This paper proposed a BCI based on P300 visually evoked potential for the control of an adult-size humanoid robot. A display of a 1 × 4 matrix was presented in front of subjects. Each stimulus represented a command for instructing the movement of humanoid robot. EEG was acquired from the Cz, Pz, and Oz channels. When subjects focused on a target, P300 components of ERP were elicited. The time-shifting correlation algorithm was proposed to resolve the uncertainty of both peak time and potential of P300. The time-shift correlation series data were collected as the inputs, and an ANN was employed to classify target stimuli and nontarget stimuli. The proposed time-shifting correlation algorithm and the ANN-based classifier were implemented on a microcontroller. To evaluate the proposed P300-based BCI, five healthy subjects were first trained via a BCI-robot simulator. Then, the online-operating sessions with an adult-size humanoid robot were performed. The performance of proposed P300-based BCI, including accuracy rate and ITR, was evaluated. Moreover, ground truth trajectories, which were collected via a motion capture system, were reported to discuss the feasibility of the proposed system. The system architecture is depicted in Figure 1.

This paper is organized as follows: Section 2 describes the method of acquisition of the EEG, Section 3 describes the proposed P300-based BCI and the integration of BCI and an adult-size humanoid robot, Section 4 reports the results and evaluations, and conclusions are drawn in Section 5.

## 2. Method

### 2.1. Subject

Five healthy subjects composed of 4 males and 1 female (mean age 22 years, standard deviation three years) participated in experiments. All subjects were students of National Taiwan University of Science and Technology and had no previous experience operating a P300-based BCI. All subjects have normal or corrected-to-normal vision.

### 2.2. Stimulus and Experimental Paradigm

**Figure 2:**
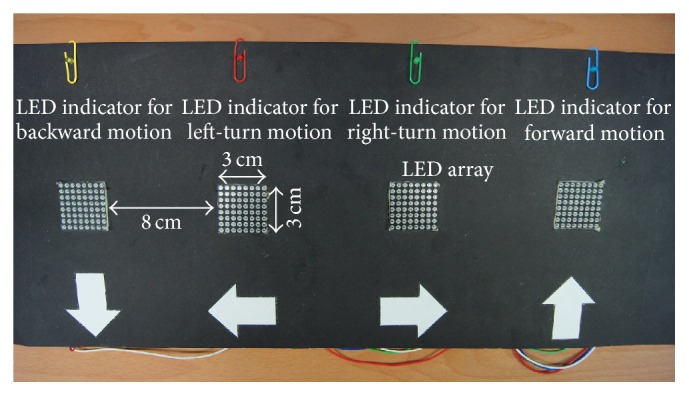
A 1 × 4 stimuli display.

Five subjects were asked to view a display of a 1 × 4 matrix, as shown in Figure 2. The display was composed of four LED array modules. The four LED array modules with the same dimensions of 3 cm × 3 cm were placed at intervals of 8 cm. Each LED array represented a command for instructing the movement of the humanoid robot. From right to left in the display, they were forward, right-turn, left-turn, and backward. Each LED array presented visible red light of moderate intensity on a black background. An LED indicator was affixed over each LED array providing the target stimulus feedback. There was a 50 cm distance between the display and the subjects, who were in comfortable postures.

**Figure 3:**
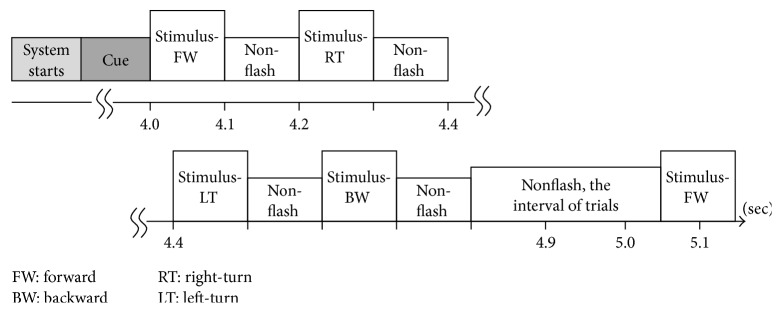
The experimental trial paradigm.

The system was triggered after subjects blinked three times, and the stimulus cue was 4 seconds. The four LED indicators flashed once from right to left during the stimulus cue. From right to left, each LED array flashed for 100 milliseconds, and the interstimulus interval was 100 milliseconds [19, 20]. An interval of 250 milliseconds passed between trials. Therefore, the total duration of a trial was 1050 milliseconds. The experimental trial paradigm is illustrated in Figure 3.

### 2.3. Data Acquisition and Preprocessing

**Figure 4:**
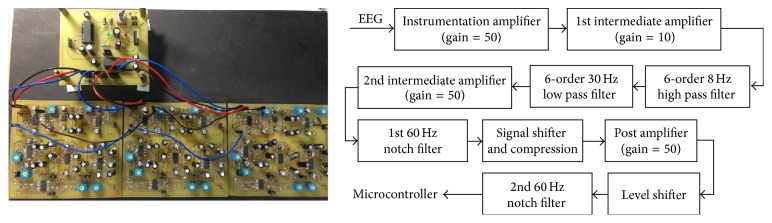
Developed EEG measurement circuit (LHS) and signal processing flowchart (RHS).

**Figure 5:**
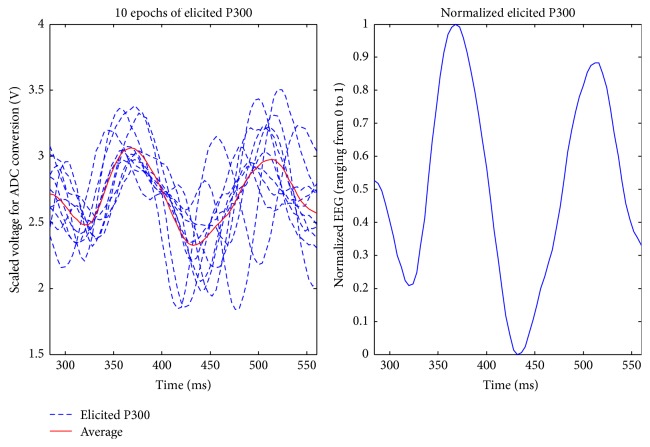
Ten elicited P300 responses and the normalized result ranging from *t* + 284 ms to *t* + 560 ms.

Reported by [9], P300 increases in amplitudes from frontal to parietal areas. In this work, electrodes placed at parietal and occipital lobes were used to measure the response EEG of P300. Three electrodes were placed at Cz, Pz, and Oz, according to the 10/20 system. A reference electrode was placed on one side of the lobes, and a ground electrode was placed on the subject's forehead. An EEG measurement circuit, as shown in Figure 4, was used to acquire EEG signal. The three EEG channels were amplified by using a 125,000 gain amplifier with an 8–30 Hz band-pass filter and sampled at 250 Hz. A 60 Hz notch filter was used online to remove environmental noise. Acquired EEG signals from Cz, Pz, and Oz were averaged as an input for further analysis of time-shifting correlation. The time-shifting correlation algorithm is based on analyzing the correlation between elicited P300 and an acquired EEG. Therefore, a database for each subject was established in the training procedure. Before starting training procedures, subjects selected a target from the display. Subjects were asked to focus on a selected target which continuously flashed at 5 Hz. Later analysis of offline row data proceeded to select the elicited P300 manually. For example, if a subject selected a target “RT,” he/she was asked to pay attention to the selected target. When a trial began at time *t*, the intervals of elicited P300 with right to left stimuli usually appeared at *t* + 81 ms to *t* + 360 ms, *t* + 281 ms to *t* + 560 ms, *t* + 481 ms to *t* + 760 ms, and *t* + 681 ms to *t* + 960 ms (*t* + 200 · *k* + 81 to *t* + 200 · *k* + 360, *k* = 0,1, 2,3). In this example, the selected target, “RT,” was the second stimulus. Therefore, the interval of *t* + 284 ms to *t* + 560 ms was collected. An average of ten elicited P300 epochs was stored in the database for a particular subject. The elicited P300 responses from the database of one subject are shown in Figure 5. Because of the variation in the amplitude and the difference in voltage levels, EEG signals were normalized from 0 to 1.

### 2.4. Time-Shifting Correlation Algorithm

The Pearson product-moment correlation coefficient (PPMCC) described the linear correlation between two variable sets, *P* and *Q*, where *P* was an acquired EEG and *Q* was elicited P300 as [21]. Equation (1) gave a feature vector, *r*, and elements ranged from 1 to −1, where 1 represented total positive correlation, 0 represented no correlation, and −1 represented total negative correlation. Because each epoch is 280 ms long, there are 70 sampled points for *P* and *Q*:

(1) ri=∑Pi−P¯×Qi−Q¯∑j=170Pj−P¯2×∑j=170Qj−Q¯2i=1,2,…,31.

**Figure 6:**
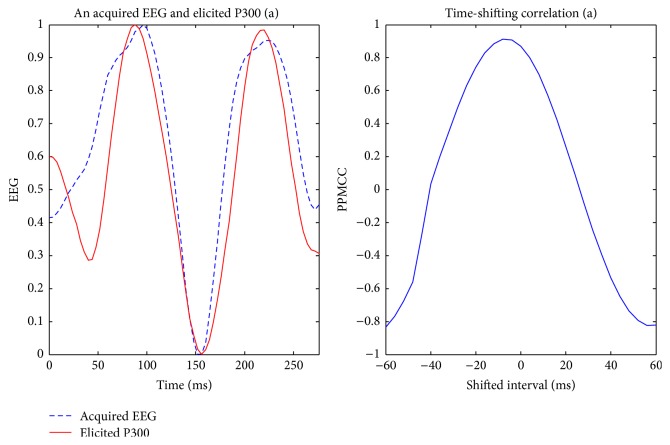
Elicited P300 and a target EEG signal ranging from *t* + 81 ms to *t* + 360 ms (LHS) and the time-shifting correlation between them (RHS).

**Figure 7 fig7:**
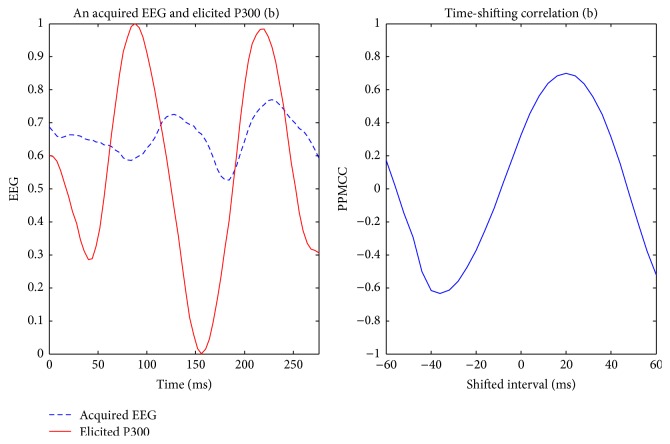
Elicited P300 and a nontarget EEG signal ranging from *t* + 281 ms to *t* + 560 ms (LHS) and the time-shifting correlation between them (RHS).

**Figure 8 fig8:**
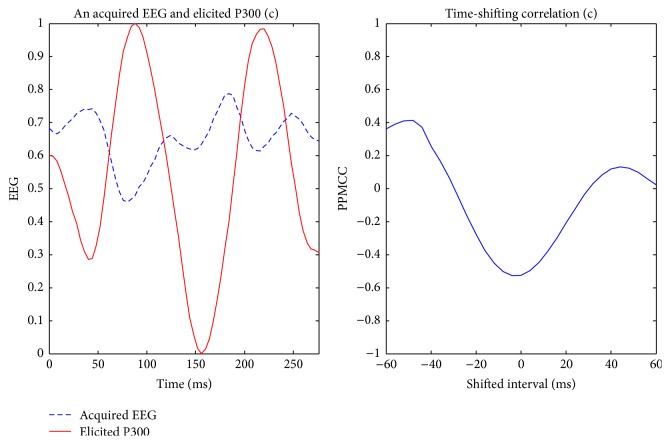
Elicited P300 and a nontarget EEG signal ranging from *t* + 481 ms to *t* + 760 ms (LHS) and the time-shifting correlation between them (RHS).

**Figure 9 fig9:**
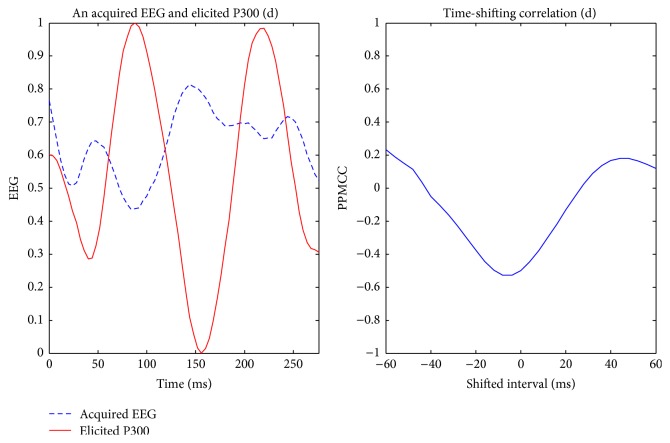
Elicited P300 and a nontarget EEG signal ranging from *t* + 681 ms to *t* + 960 ms (LHS) and the time-shifting correlation between them (RHS).

**Figure 10:**
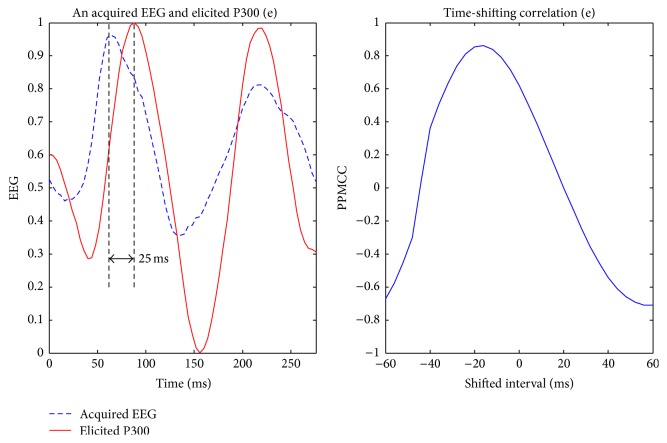
Elicited P300 and a target EEG signal with shifting latency (LHS) and the time-shifting correlation between them (RHS).

Visually evoked potential occurs with a latency of 250 milliseconds to 500 milliseconds between stimulus and response. Moreover, the elicited time and the amplitude of the P300 are related to visual fatigue of subjects, especially after long-term operation. Therefore, the time-shift correlation was used to resolve peak time uncertainty in the P300. An acquired EEG was shifted forward and backward within the range of 60 milliseconds with a shifting interval of 4 milliseconds for computing each correlation. Hence, there were 31 PPMCCs associated with each acquired EEG. In Figures 6 [Fig fig7] [Fig fig8] [Fig fig9]–10, elicited P300 from the database and five acquired EEG signals are shown. They were all normalized and ranged from 0 to 1. In Figure 6, there was a high correlation between the acquired EEG and the elicited P300. After the system computed the time-shifting correlation, 31 PPMCCs were obtained and presented a bell-shaped curve when plotted. In contrast, in Figures 7 [Fig fig8]–9, because there were low correlations between the acquired EEG signals and the elicited P300, the time-shifting correlation presented irregular sine curves.  Figure 10 shows an acquired EEG which correlated well with the elicited P300, but there was a shifting interval between the two. However, the time-shifting algorithm was robust to time uncertainty, and a bell-shaped curve was obtained.

### 2.5. Feedforward Backpropagation Neural Network for BCI Classification

**Figure 11:**
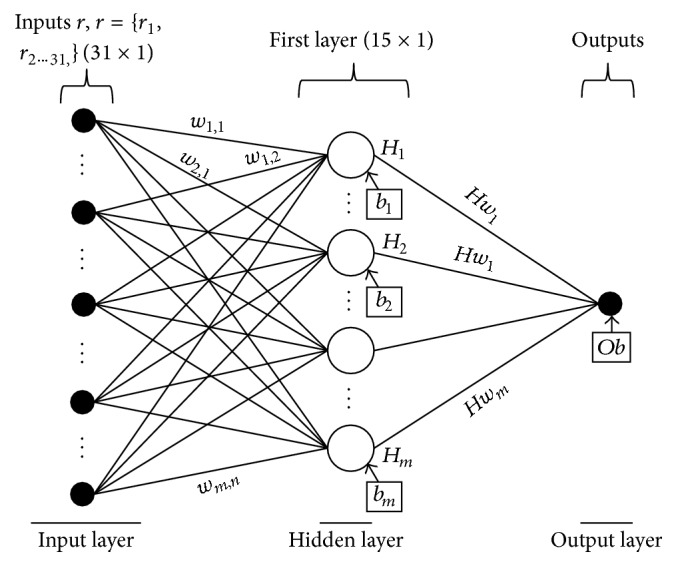
The architecture of the employed FFNN.

ANNs are computational models that are inspired by biological nervous systems. They mimic the brain's mechanism as a nonlinear and continuous-time system to simulate intelligence. The feedforward neural network (FFNN) is one of the most common architectures and is used for classification in this paper. Known as a multilayer perceptron, an FFNN consists of a number of neurons that are organized in layers, including an input layer, hidden layers, and an output layer. A single-hidden-layer FFNN with one output was adopted, as shown in Figure 11. Specifically, node outputs were expressed via (2). In this work, the FFNN toolbox provided in MATLAB was used to train classifier models. The numbers of hidden layers and neurons were determined according to trial and error method:

(2) Ni=∑j=1nxj·wi,j+bii=1,2,…,m,Hi=fNii=1,2,…,m,y=∑i=1mHi·Hwi+Ob.

*x* is the input vector which includes 31 PPMCCs. *n* and *m* are the number of inputs and hidden neurons, where *n* = 31 and *m* = 15. *w* and *Hw* are the input and hidden weights. *b* is the bias vector of hidden layers, and *Ob* is the bias of an output layer. As mentioned in [22], the hyperbolic tangent sigmoid function is used as the activation function of a neural network, as shown in

(3) $$\begin{array}{c} f\left(\;N_{i}\;\right)=\frac{2}{1+\exp\left(-2\times N_{i}\right)}-1. \end{array}$$

The backpropagation algorithm was deployed for the neural network training. It was assumed that a given training dataset included *p* input-target sets. The sum of squared errors *E* is shown in

(4) $$\begin{array}{c} E=\overset{p}{\underset{k=1}{\sum }}{\left(\;T_{k}-y_{k}\;\right)}^{2}. \end{array}$$

When *E* is equal to zero, a neural network processes exactly one input to each desired target. Hence, network variables, including *w*, *Hw*, *b*, and *Ob*, are adjusted to minimize the squared error by applying a backpropagation algorithm. There were *m* × (*n* + 2) + 1 variables arranged in a deployed FFNN that should be updated through the backpropagation algorithm.

**Figure 12:**
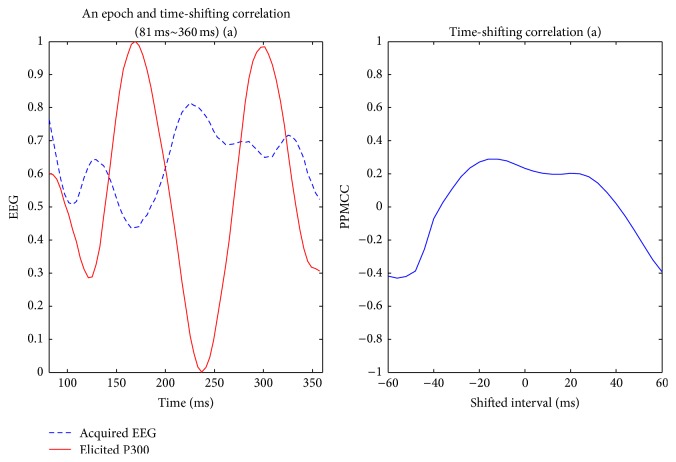
The first interval, *t* + 81 ms to *t* + 360 ms, of acquired EEG and the time-shifting correlation diagram; output value = 0.2910.

**Figure 13 fig13:**
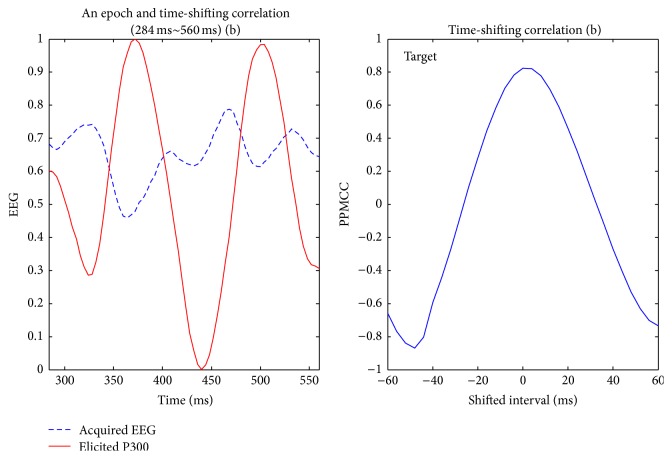
The second interval (target), *t* + 284 ms to *t* + 560 ms, of acquired EEG and the time-shifting correlation diagram; output value = 0.9473.

**Figure 14 fig14:**
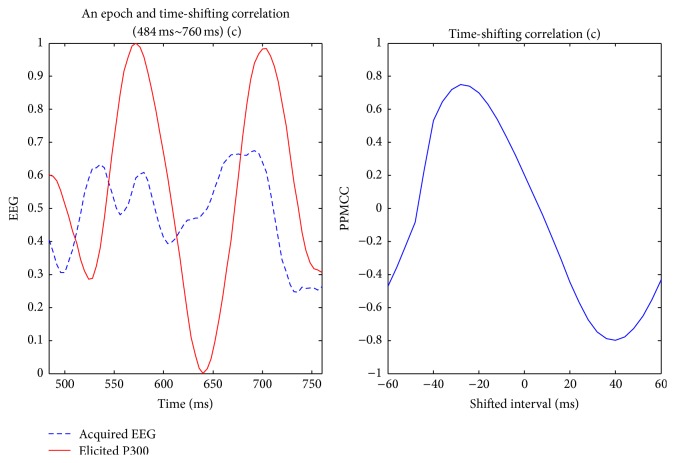
The third interval, *t* + 484 ms to *t* + 760 ms, of acquired EEG and the time-shifting correlation diagram; output value = 0.2493.

**Figure 15:**
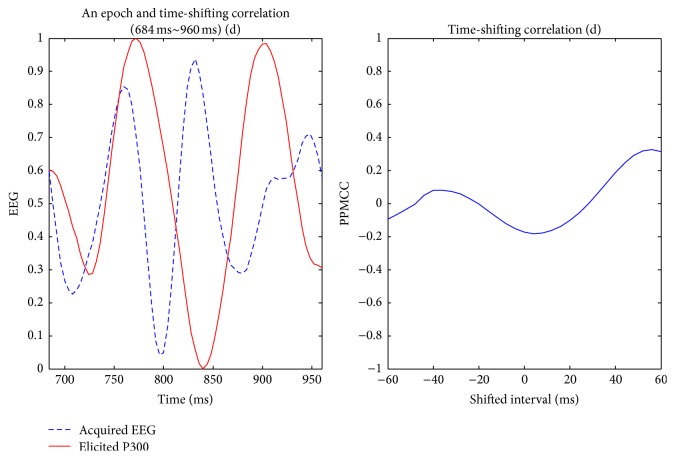
The fourth interval, *t* + 684 ms to *t* + 960 ms, of acquired EEG and the time-shifting correlation diagram; output value = 0.0233.

**Figure 16:**
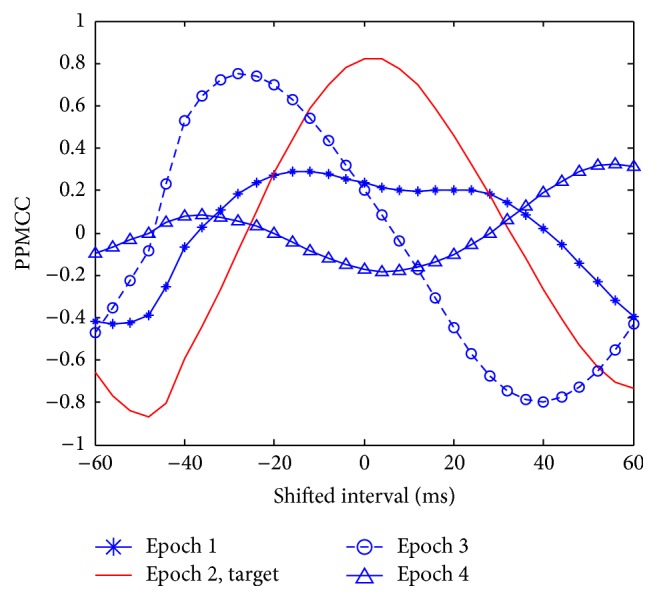
Time-shifting correlations for the four intervals.

After the subjects blinked three times, the system started and followed the protocol depicted in Figure 3. A 280-millisecond window was applied to detect possible P300 potential with an averaged amplitude of the three selected channels. The window moved in time, and the time-shifting correlation algorithm was activated to obtain 31 PPMCC features. These features were imported to the trained FFNN model, which generated an output for each epoch, and the stimulus was judged to be either a target or a nontarget according to the output. Target or nontarget stimuli were classified by a defined threshold which was determined in experiments. The threshold was 0.6 in this paper, and the details for selecting a proper threshold are discussed later in Section 4.1. In Figures 12 [Fig fig13] [Fig fig14]–15, acquired EEG signals of four intervals in a trial are depicted, and the elicited P300 occurs at the interval of *t* + 284 ms to *t* + 560 ms. A bell-shaped curve was observed in Figure 13. Then, features for four intervals, as shown in Figure 16, were imported into the trained FFNN. The outputs for the four feature sets were 0.2910, 0.9473, 0.2493, and 0.0233. Because the threshold was set at 0.6, a target was obtained from the second stimulus.

## 3. P300-Based BCI

P300 signals were prominent in the parietal and occipital electrodes. However, their amplitudes ranged from 10 *μ*V to 25 *μ*V, which were easily affected by background noise. Usually, the numbers of epochs associated with target and nontarget stimuli are averaged to ensure reliable detection. As a result, the number of averages decreases the ITR of P300-based BCIs. Hence, this paper proposes two methods, FM and AM, which were adapted for different desired purposes. In this work, a target epoch is obtained when the output of the FFNN is higher than the defined threshold. Legal trials were defined as when only an output of the FFNN is higher than the defined threshold. Multiple targets and nontargets are regarded as illegal trials.

### 3.1. Fast-Recognized Mode (FM)

**Figure 17:**
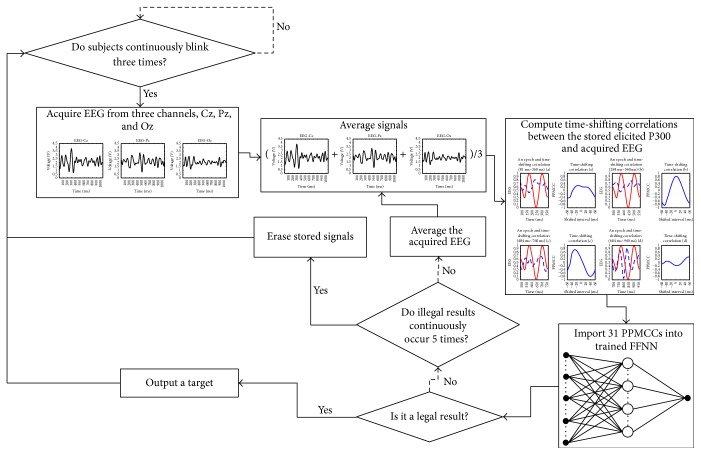
Block diagram of the FM.

The block diagram of FM is shown in Figure 17. The FM recognized either targets or nontargets by the last legal trial. When a legal result was obtained, when an output of the FFNN was higher than the defined threshold, the system consequently generated a command to the robot according to the selected target. However, for an illegal result, such as multiple targets and nontargets, the epoch was stored and averaged with the next epoch. Therefore, the features of elicited P300 became more distinct and recognition improved. The maximum number of averaged epochs was 5. After 5 trials, if no legal result was obtained, the stored epochs were erased.

### 3.2. Accuracy-Recognized Mode (AM)

**Figure 18:**
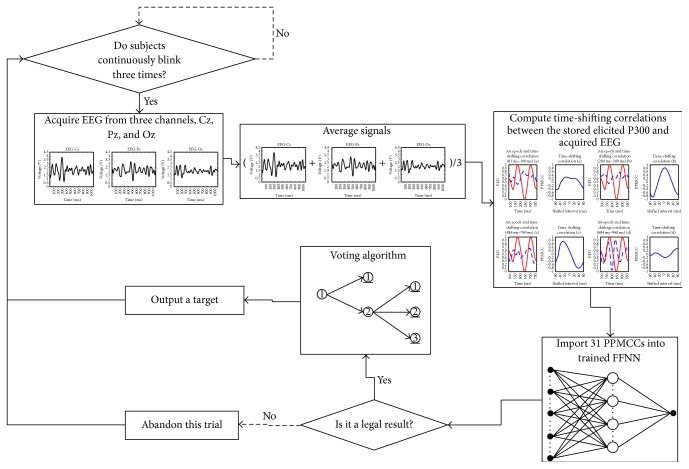
Block diagram of the AM.

**Figure 19:**
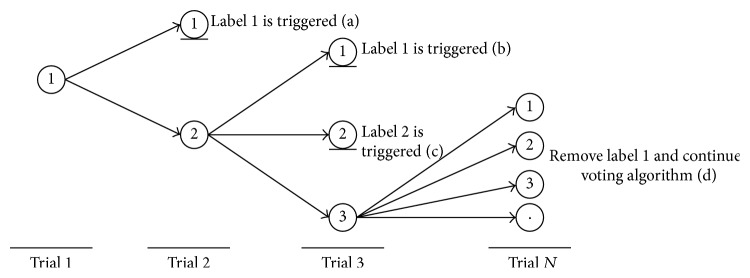
The voting algorithm of AM.

The AM was accomplished via a voting algorithm. A target was obtained when the same result occurred twice in the last three legal trials. The block diagram of AM is depicted in Figure 18. For example, the voting algorithm begins as a target is triggered, as shown in Figure 19. The triggered target is assumed to be labeled “1,” and this legal trial is called the first trial. When the target labeled “1” is triggered again in the second legal trial, the target labeled “1” is obtained as route (a). However, if a target labeled “2” which is different from label “1” is triggered in the second legal trial, the voting algorithm proceeds to the third legal trial. In the third legal trial, if the target labeled “1” is triggered, the target labeled “1” is obtained as route (b); if the target labeled “2” is triggered, the target labeled “2” is obtained as route (c). If a different target labeled “3,” which is different from labels “1” and “2,” is triggered, the result of the first trial is abandoned. Targets are then composed of the second, the third, and the next legal trials as route (d). The procedure is repeated until a target is obtained according to the rules of the voting algorithm.

### 3.3. Averaging Method

Averaging methods, which average several epochs to achieve high accuracy performance, are common techniques in P300-based BCIs [16]. In this paper, different quantities of epochs, including one, two, and three, were implemented. Four intervals were observed for the potential existence of an elicited P300, and the time-shifting correlation algorithm was activated to obtain 31 PPMCC features as well. The 31 PPMCC features as an input vector were fed into a trained FFNN. The four outputs that were generated by a trained FFNN represented either targets or nontargets. A legal result was obtained when the output of the FFNN was higher than the defined threshold.

### 3.4. Online Operation for Adult-Size Humanoid Robot Control

Online operations were arranged in a 7 m × 2 m area with moderate lighting. A desired trajectory was indicated with black tape, and the robot started at one end. Subjects were asked to sit behind the active area and were presented with a stimulus display. They were requested to instruct the robot to follow the desired trajectory as closely as possible. A motion capture system was introduced to record ground truth trajectories. An optical sensor with lightening red LED module was affixed to the hip of the robot. Different gait lengths under different modes, FM and AM, were further examined. However, before the online-operating sections, a BCI-robot simulator was developed to provide an efficient way to evaluate the performance of different modes.

## 4. Experiment

### 4.1. FFNN Training

Each subject performed 160 trials, which were labeled targets and nontargets, to train FFNNs. Subjects were instructed to focus on a particular target according to an arranged sequence, and each target was performed 40 times. At the beginning of each trial, the time-shift correlations for four intervals were obtained. In these four epochs, there were three nontargets and one target. Input vectors (time-shift correlations) and output vectors (targets or nontargets) for epochs were randomly divided into three sets. Seventy percent of the 160 trials (112 trials) were used to train the FFNN; 15% of the 160 trials (24 trials) were independently used to test the network generalization; 15% of the 160 trials (24 trials) were used to stop training before overfitting during network generalization. The introduced FFNN based on the backpropagation learning algorithm was trained by a neural network tool box provided in MATLAB. Generally, neural network models converged after 9000 epochs with 10^−5^ of the MSE.

**Figure 20:**
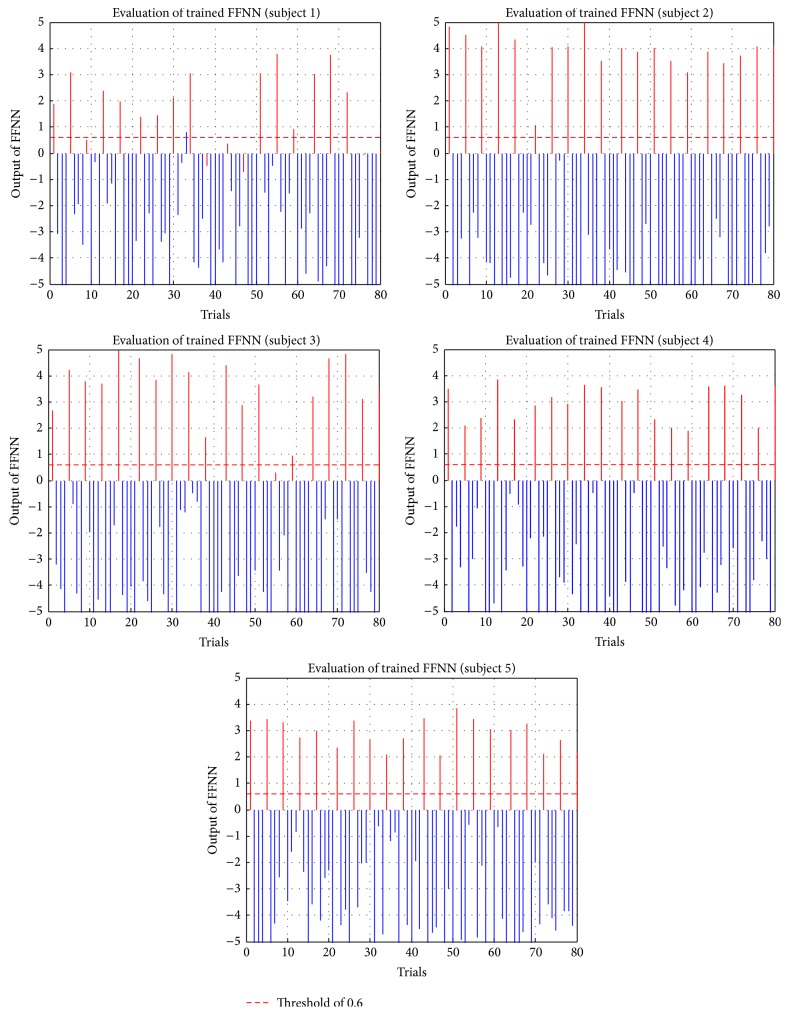
Evaluations of trained FFNN of all subjects.

To determine a threshold that classified targets or nontargets, 20 trials were further selected randomly from the 160 trials. Time-shift correlations as input vectors were imported into the trained FFNN, and the four outputs for the trial were obtained. Target epochs were labeled “1” and nontarget epochs were labeled “0,” so the four outputs described how similar they were between input vectors and the target. Evaluations of trained FFNN for the five subjects are shown in Figure 20, where the horizontal axis is the number of trials and vertical axis is the magnitude of outputs from trained FFNNs. Red solid lines and blue solid lines are targets and nontargets, respectively, which were defined according to arranged sequences. Comparing targets with nontargets, there were significant differences with respect to the magnitudes of outputs. In target epochs, outputs exceeded 0.7 in labeled targets; in nontarget epochs, outputs were usually less than 0.0. Hence, a threshold of 0.6 was defined to classify targets or nontargets of epochs.

### 4.2. The Performance of BCI

In this session, subjects were asked to follow an arranged sequence which contained 100 trials with randomly selected targets. Following the protocol, which is depicted in Figure 3, subjects focused on a target according to an arranged sequence and EEG signals were recorded. In addition to the proposed AM and FM, different quantities of one, two, and three averaged epochs were performed. Hence, 500 trials for a subject were performed for comparison between the proposed and averaging methods. Experimental results for five subjects with the different methods are listed in Table 1. In the 100 trials for each method, the numbers of correct, wrong, and nonreorganization trials were counted. Meanwhile ITRs were evaluated according to (5), where *T*_exp_ is experimental time (sec); *N*_*o*_ is the number of correctly target events; *K*_*s*_ is the number of stimuli; and *P*_*a*_ is accuracy [23]. Consider

(5) ITR=BitsCommand·60CTICTI=TexpNoBitsCommand=log2⁡Ks+Palog2⁡Pa+1−Pa×log2⁡1−PaKs−1.

The proposed FM and AM showed the highest second-highest averaged ITRs when compared to average methods. The proposed AM showed the highest averaged accuracy rate in all methods, but strict recognition constraints resulted in a lower ITR when compared to FM. In addition, the proposed FM represented the second-highest averaged accuracy rate. However, FM featured high ITRs that were capable of different desired applications. Although the averaging method with one epoch generated an output every trial, its poor averaged accuracy rate was unable to offer a friendly BCI. The averaging method with three epochs required a longer recognition time, but there was no significant improvement with respect to averaged accuracy rate.

### 4.3. Visual Fatigue Effect

In addition to comparing the accuracy and ITRs of different methods, the accumulations and the accumulation rates of wrong reorganizations were examined in this study, where the accumulation rate of wrong reorganizations was computed according to

(6) $$\begin{array}{c} Er_{i}=\frac{En_{i}-Et}{Et}\;i=1,2,\ldots ,100. \end{array}$$

**Figure 21:**
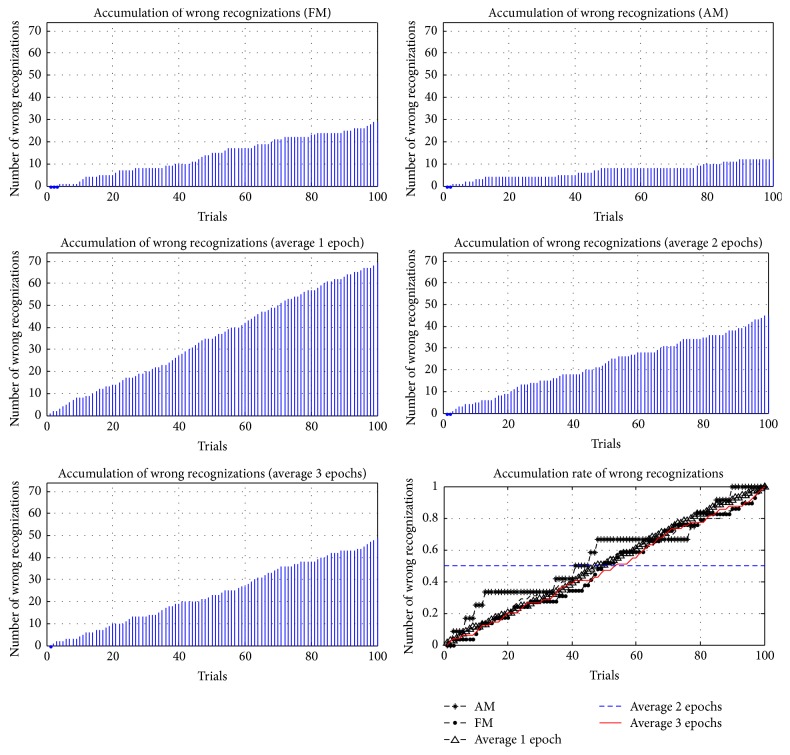
Accumulations of wrong reorganization for FM, AM, and averaging methods for subject 1.

**Figure 22 fig22:**
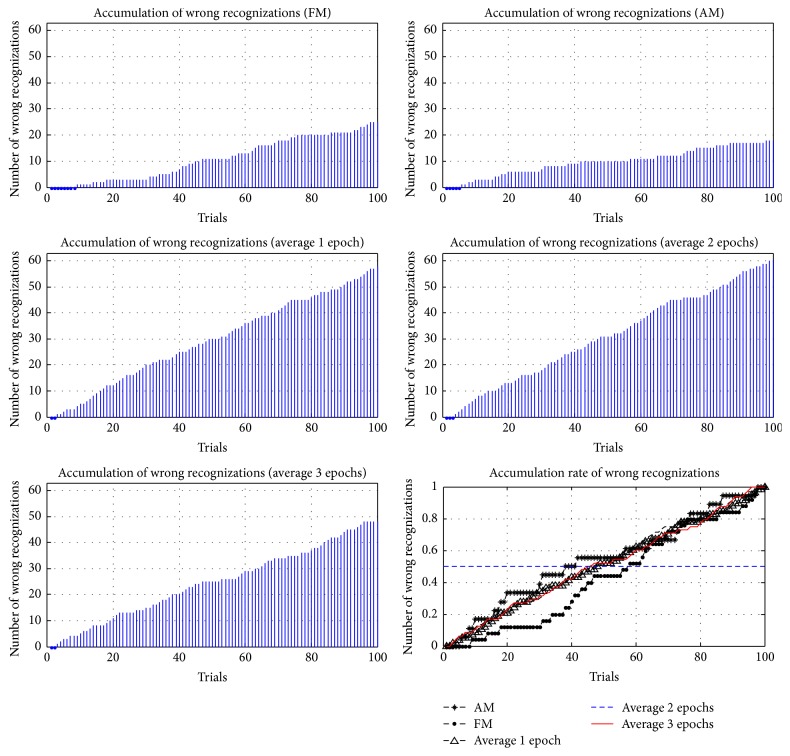
Accumulations of wrong reorganization for FM, AM, and averaging methods for subject 2.

**Figure 23 fig23:**
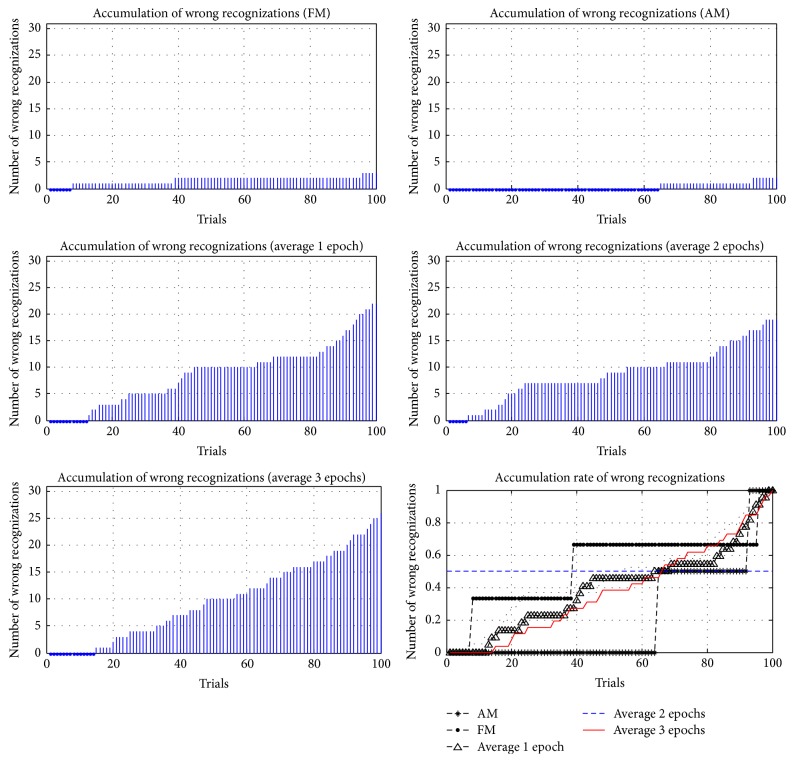
Accumulations of wrong reorganization for FM, AM, and averaging methods for subject 3.

**Figure 24 fig24:**
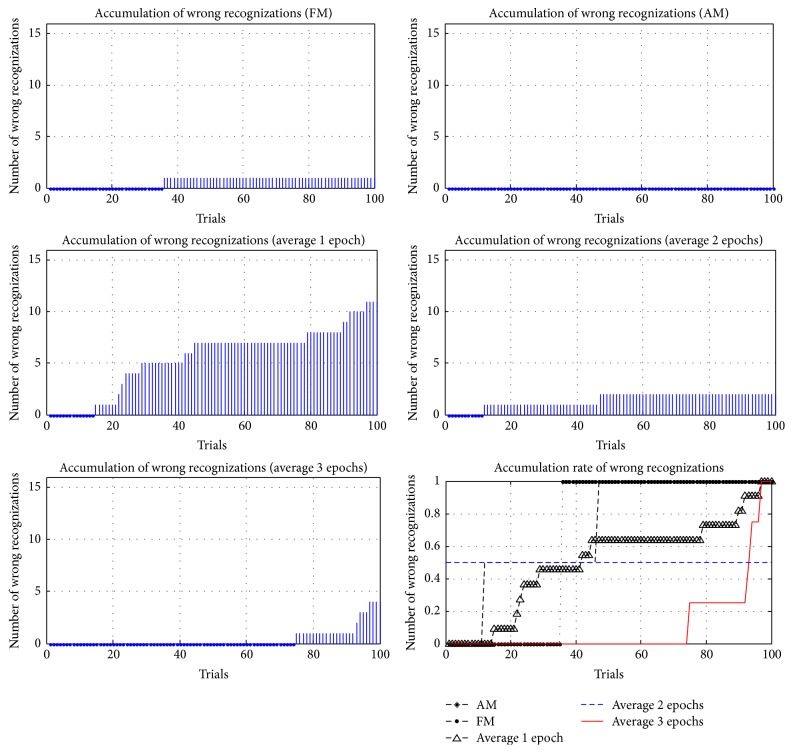
Accumulations of wrong reorganization for FM, AM, and averaging methods for subject 4.

**Figure 25:**
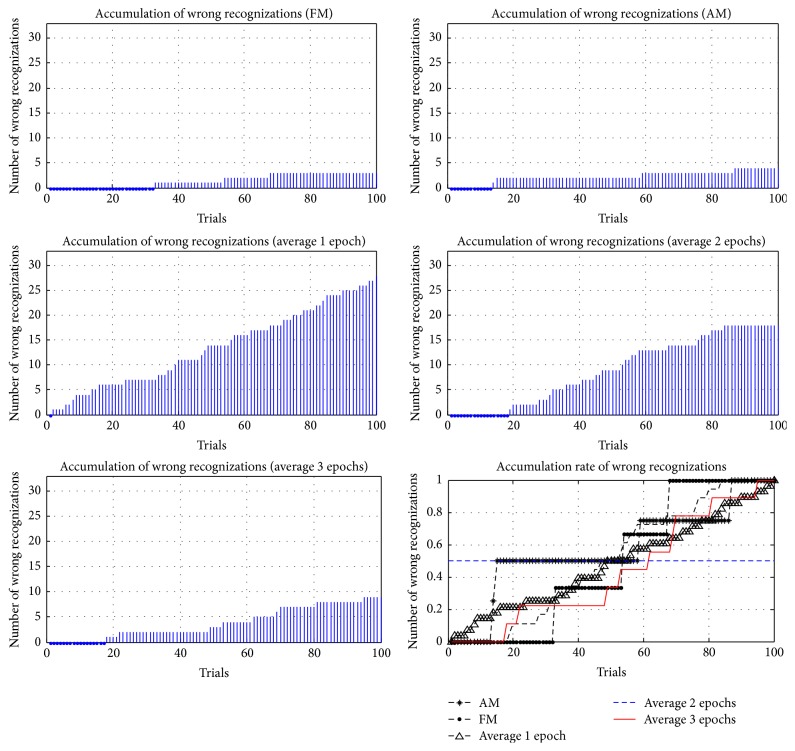
Accumulations of wrong reorganization for FM, AM, and averaging methods for subject 5.

*Er* is the accumulation rate of each trial; *En* is the cumulative number of wrong reorganizations; and *Et* is the total number of wrong reorganizations. In 100 trials for each method, the accumulations and accumulation rates of wrong reorganizations are represented in Figures 21 [Fig fig22] [Fig fig23] [Fig fig24]–25, where FM, AM, and averaging methods (1, 2, and 3 epochs) were depicted. The accumulations of wrong reorganizations and trials are indicated on the *y*-axis and the *x*-axis, respectively. In addition, a horizontal dashed line indicates an accumulation rate of 0.5. In FM, AM, and averaging methods with one epoch and two epochs, accumulation rates exceeded 0.5 after 50 trials. In the averaging method with three epochs, accumulation rates were still less than 0.5 after 50 trials. From the observations regarding accumulation rates, wrong reorganizations of FM, AM, the averaging method with one epoch, and the averaging method with two epochs were uniformly distributed in trials, and the curves of accumulation rates gradually increased. The averaging method with three epochs had the highest averaged accuracy rate of all averaging methods, but wrong reorganizations occurred in later trials. The number of wrong recognitions increased faster after 60 trials, at approximately five minutes after the first trial. The proposed AM and FM featured stable accuracy rates over long operation times and were less affected by visual fatigue effects. In contrast, the averaging method with three epochs featured the highest accuracy rate among the averaging methods, but the wrong recognition rate increased quickly with longer operating time. Thus, this averaging method was unsuitable for long operations. As operating time increased, the significant decrease in the accuracy rate is a concern for practical applications.

### 4.4. Online Operation

**Figure 26:**
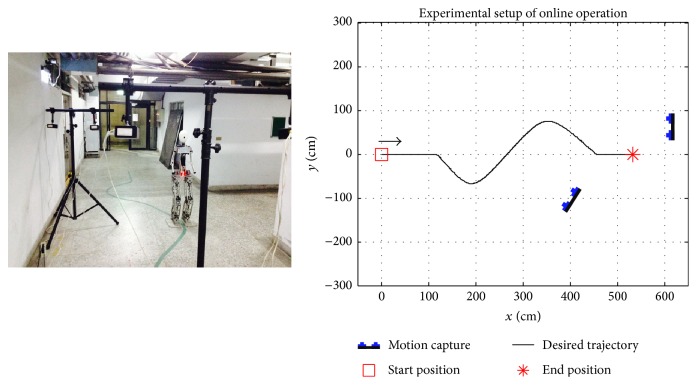
The experimental setup for online operation.

**Figure 27:**
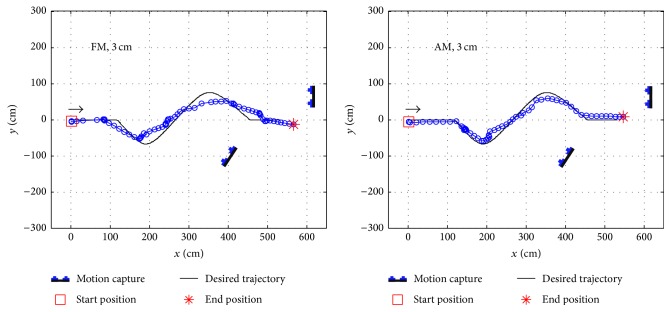
Ground truth trajectories of robot using FM, 3 cm gait length (LHS), and AM, 3 cm gait length (RHS).

The experimental setup is shown in Figure 26. In each trial, the operating mode (AM or FM) and a fixed gait length (3 cm or 6 cm) of the robot were set. Therefore, subjects were unable to switch the operating mode or change the speed of the robot. Each subject performed four trials including 3 cm and 6 cm gait lengths.  Figure 27 (LHS) shows the ground truth trajectory of a robot, where blue circles were the position of the robot's hip at each captured frame. To record ground truth trajectories clearly from a motion capture system, an optical LED maker was affixed to the hips' center of the robot. In Figure 26, the red light was an optical marker, and the trajectory of the LED maker represents the ground truth trajectories of the robot. A low pass filter was used to smooth the recorded trajectories. The finish time, total length, PPMCC, and length difference are listed in Table 2. In AM, when a 3 cm gait length was set, the robot's trajectories featured the highest averaged PPMCC, which signified that there were high correlations between the robot's and the desired trajectories. The robot's trajectory closely matched the desired trajectory. AM featured the highest accuracy rates with a longer response time. Although there were low ITRs with AM, the robot's trajectories could be corrected under a lower walking speed when errors occurred. In FM, when a 3 cm gait length was set, the averaged PPMCC decreased but it still represented high correlations between the robot's and the desired trajectories. FM featured higher ITRs but a lower accuracy rate when compared to AM. However, with the lower accuracy rates of FM, the robot's trajectories could be corrected with higher ITRs when errors occurred.

In AM, when a 6 cm gait length was set, the lowest averaged PPMCC occurred that showed there were low correlations between the robot's trajectory and the desired trajectory. When errors occurred, the direction of the robot could not be corrected in time due to the low ITR of AM. However, in FM, when a 6 cm gait length was set, the averaged PPMCC was improved and the shortest finishing time was measured. Although the robot was operating at a higher speed, the high ITR of FM was also able to respond faster to the robot's directions and correct them when errors occurred. When AM was selected, a lower walking speed of the robot was recommended, especially in a crowded area, for higher accuracy of recognition to reduce the likelihood of collision. When FM was selected, a higher walking speed was possible when navigating a robot efficiently in a spacious area. FM was also used to respond faster to the uncertainty in the environment.

### 4.5. Performance Comparison with Other Approaches

In order to provide performance comparison of our approach with other conventional approaches, canonical correlation analysis of (CCA) spatial filter and support vector machine (SVM) was additionally done for comparisons with new processed experiments. The results of averaging 1 to 3 times epochs from two classifiers of three subjects were summarized in Table 3. The results demonstrated that the proposed BCI outperformed higher performance in 1-epoch experiment with lower illegal rate than the CCA SVM classifier. Although the accuracy rates in average 2 and 3 epochs are similar to the CCA SVM classifier, the ITRs of these two subjects were significantly improved. Notably, the ITR is very crucial to the applications of real-time control purposes because a higher ITR means a faster command update rate and a greater control resolution. Hence, the proposed approach is very suitable for real-time control purposes, especially for online humanoid locomotion control.

## 5. Conclusions

BCIs have been widely discussed and show a great potential for providing subjects with a nonmuscular method to connect with world. A P300-based BCI for the navigation of an adult-size humanoid robot was proposed in this paper. A P300-based system has the advantages of using a small amount of a subject's data for training and modeling. However, visually evoked potential occurs with a latency of 250 milliseconds to 500 milliseconds between stimulus and response. Moreover, the elicited time and the amplitude of the P300 relate to the visual fatigue of subjects. Therefore, a time-shifting algorithm was proposed. The proposed time-shifting algorithm was activated to obtain four sets of 31 PPMCC features for four intervals where the elicited P300 usually appeared after a stimulus in the proposed system. The 31 PPMCC features for each interval were collected as the inputs, and a trained FFNN was employed to classify target and nontarget stimuli.

Two operating modes (FM and AM) were implemented for different conditions. An adult-size humanoid robot was introduced to perform online-operating sessions, and motion capture was deployed to record ground truth trajectories. With respect to conditions of navigation, the operating modes and the walking speeds of the robot were examined. AM featured high accuracy rates and was suitable for high accuracy of recognition to reduce the likelihood of collision; FM featured high ITRs and was able to navigate the robot efficiently to explore a spacious area. A hybrid BCI that integrates event related potential (ERP) and motor imagery (MI) is a solution to meet the task of higher uncertainty in robot navigation. In addition, a P300-based BCI is expected to multitask in the future, including the operation of upper limbs of a robot.

In the future, an optimal NN approach will be applied to this study in the future to resolve the drawback of using trial-and-error selection of NN model parameters in this paper, as well as improving the performance of NN model. Moreover, semiautonomous navigation function with visual perception for humanoid robot will be done to avoid the obstacles in front of the robot so that the proposed BCI approach could be more practical and feasible to be used in the daily life.

## Figures and Tables

**Table 1:** Evaluation of BCI.

Methods	Correct/wrong/nonreorganization rate (%)					
	Subject 1	Subject 2	Subject 3	Subject 4	Subject 5	Average
Fast mode, FM	71/0/29	97/3/0	75/18/7	99/1/0	97/3/0	**87.8/5.0/7.2**
Data transfer rate (bit/min.)	8.54	70.61	15.72	101.58	67.20	**52.73**

Accuracy mode, AM	88/12/0	96/4/0	78/22/0	100/0/0	98/2/0	**92.0/8.0/0.0**
Data transfer rate (bit/min.)	10.24	40.24	9.91	52.42	43.53	**31.27**

Average 1 epoch	31/8/61	72/0/28	42/9/49	89/0/11	78/4/18	**62.4/4.2/33.4**
Data transfer rate (bit/min.)	0.23	28.83	2.38	67.42	39.72	**27.72**

Average 2 epochs	55/2/43	83/0/17	40/4/56	98/0/2	81/1/18	**71.4/1.6/27.0**
Data transfer rate (bit/min.)	4.62	25.44	0.89	51.15	23.08	**21.04**

Average 3 epochs	51/0/49	91/0/9	52/2/46	96/0/4	74/1/25	**72.8/0.6/26.6**
Data transfer rate (bit/min.)	2.17	24.63	2.38	30.98	10.73	**14.18**

**Table 2:** Experimental results of online operation.

Mode (gait length)	Subject #						
	Subject 1	Subject 2	Subject 3	Subject 4	Subject 5	Average	STD
*Fast mode, FM (3 cm)*							
Finishing time (sec.)	255.00	245.00	238.00	283.00	257.00	**255.60 **	**17.14 **
Total length (cm)	660.40	639.50	606.60	716.80	668.00	**658.26 **	**40.46 **
PPMCC	0.40	0.42	0.62	0.41	0.43	**0.46 **	**0.09 **
Length difference (%)	1.60%	−1.62%	−6.68%	10.28%	2.77%	**1.27%**	**—**

*Accuracy mode, AM (3 cm)*							
Finishing time (sec.)	249.00	228.00	228.00	263.00	235.00	**240.60 **	**15.18 **
Total length (cm)	638.50	672.00	625.10	726.30	697.90	**671.96 **	**41.64 **
PPMCC	0.56	0.58	0.49	0.43	0.49	**0.51 **	**0.06 **
Length difference (%)	−1.80%	3.40%	−3.80%	11.70%	7.40%	**3.38%**	**—**

*Fast mode, FM (6 cm)*							
Finishing time (sec.)	191.00	165.00	165.00	166.00	170.00	**171.40 **	**11.15 **
Total length (cm)	580.20	631.50	703.50	704.28	724.62	**668.82 **	**60.87 **
PPMCC	0.48	0.59	0.46	0.24	0.36	**0.43 **	**0.13 **
Length difference (%)	−10.70%	−2.80%	8.20%	8.40%	11.50%	**2.92%**	**—**

*Accuracy mode, AM (6 cm)*							
Finishing time (sec.)	161.00	142.00	118.00	172.00	175.00	**153.60 **	**23.73 **
Total length (cm)	667.40	600.30	595.30	729.10	730.80	**664.58 **	**66.11 **
PPMCC	0.10	0.43	0.45	0.49	0.31	**0.36 **	**0.16 **
Length difference (%)	2.70%	−7.60%	−8.40%	12.20%	12.40%	**2.26%**	**—**

**Table 3:** Performance comparison of the proposed approach and conventional CCA SVM.

Methods	Classification accuracy/illegal trial rates (%)					
	Subject 1		Subject 2		Subject 3	
	Proposed BCI	CCA SVM	Proposed BCI	CCA SVM	Proposed BCI	CCA SVM
Average 1 epoch	90.0/30.0	87.92/40.0	84.81/21.0	83.52/46.0	75.00/28.0	74.00/50.0
Data transfer rate (bit/min.)	49.41	38.49	43.82	28.17	24.45	16.09

Average 2 epochs	95.35/14.0	96.22/15.50	88.64/12.0	91.44/18.50	83.72/14.0	87.1/19.0
Data transfer rate (bit/min.)	38.77	39.68	29.18	30.72	22.65	25.01

Average 3 epochs	100.0/3.03	100.0/7.0	96.67/9.09	97.78/9.25	90.0/9.09	90.48/12.0
Data transfer rate (bit/min.)	36.57	35.07	28.78	30.30	21.18	20.95

Accuracy rate = correct/(correct + incorrect).

Illegal trial rate = illegal/total trials.
