# Corrigendum to “Time-Shift Correlation Algorithm for P300 Event Related Potential Brain-Computer Interface Implementation”

**DOI:** 10.1155/2017/8012547

**Published:** 2017-04-05

**Authors:** Ju-Chi Liu, Hung-Chyun Chou, Chien-Hsiu Chen, Yi-Tseng Lin, Chung-Hsien Kuo

**Affiliations:** ^1^Department of Internal Medicine, School of Medicine, College of Medicine, Taipei Medical University, Taipei 110, Taiwan; ^2^Division of Cardiology, Department of Internal Medicine, Shuang Ho Hospital, New Taipei City 235, Taiwan; ^3^Department of Electrical Engineering, National Taiwan University of Science and Technology, Taipei 106, Taiwan; ^4^Graduate Institute of Biomedical Engineering, National Taiwan University of Science and Technology, Taipei 106, Taiwan; ^5^Department of Biomedical Engineering, National Defense Medical Center, Taipei 114, Taiwan

In the article titled “Time-Shift Correlation Algorithm for P300 Event Related Potential Brain-Computer Interface Implementation” [1], an acknowledgment should be added as follows:

The authors would like to thank the Ministry of Science and Technology (under Grant MOST 104-2221-E-011-140) and the Taipei Medical University/National Taiwan University of Science and Technology cross-university collaboration project (under Grant TMU-NTUST-103-07) for partial support of this work.
